# The association of cervical cancer screening and quality of care: A systematic analysis of the Global Burden of Disease Study 2019

**DOI:** 10.7189/jogh.13.04090

**Published:** 2023-08-25

**Authors:** Minmin Wang, Yinzi Jin, Zhi-Jie Zheng

**Affiliations:** 1 Department of Global Health, School of Public Health, Peking University, Beijing, China; 2 Institute for Global Health and Development, Peking University, Beijing, China

## Abstract

**Background:**

Improving the quality of care is vital to enhance outcomes for cervical cancer patients. However, the inequality of cervical cancer care was seldomly assessed.

**Methods:**

We collected the data of cervical cancer burden from the Global Burden of Disease 2019 database, and constructed the Quality of Care Index (QCI) using principle component analysis. Then the disparity of QCI across regions and populations were evaluated. The association between cervical cancer screening coverage and QCI weas also explored.

**Results:**

Quality of cervical cancer care was of disparity across regions with different development levels, with a widening gap between low-income regions and others. Cervical cancer QCI dropped rapidly after the age of 35. Cervical cancer screening coverage was positively associated with QCI, and this association was stronger in countries with low- and middle-development levels.

**Conclusions:**

Regions with a low development level and the middle-aged women were vulnerable in QCI improvement. Higher screening coverage was associated with better cervical cancer QCI, implying that expanding cervical cancer screening coverage may be an effective strategy to improve the quality of cervical cancer care.

Cervical cancer is the second most common cancer and the second most common cause of death from cancer in women of reproductive age worldwide [1]. In developing countries, cervical cancer ranks the second most common and lethal tumours, while it ranks the tenth in developed countries [2], making cervical cancer an example of global inequality [3,4]. It is estimated that by 2040, there will be 855 130 new cases and 533 588 deaths of cervical cancer globally, among which 161 662 cases and 112 258 deaths would occur in regions with low development levels and bringing a huge health and economic burden [5].

Global strategies to combat cervical cancer as a global health concern have been established. In May 2018, the Director-General of the World Health Organization (WHO) announced a global call of action to eliminate cervical cancer as a public health problem [6]. Strategies and roadmaps have been proposed to achieve the global goal [7-9]. Human papillomavirus (HPV) vaccination, screening and treatment of precancerous lesions, and cancer management are the three pillars of cervical cancer prevention [10], and clear target have been set to monitor the progress of cervical cancer elimination for the period 2020-2030 [11].

The quality of care refers to providing timely and professional healthcare services to achieve the desired health outcomes. Improving the quality of care is a key measure to improve health outcomes. Model simulations have shown that improving the quality of care can increase the five-year net survival rate of cervical cancer patients worldwide from 42.1% to 57.5% [12]. Thus, quality of care is essential in reducing cervical cancer burden to achieve the global goal of cervical cancer elimination. However, the current status and the inequality of quality of care in cervical cancer has not been well evaluated.

In this study, we assessed the data from Global Burden of Disease (GBD) 2019 and established the Quality of Care Index (QCI) to evaluate the quality of care in cervical cancer. It is assumed that cervical cancer screening could improve the quality of cervical cancer care, which was explored through association analysis. Results from the current study would provide evidence to inform the vulnerable population and possible strategies in improving cervical cancer quality of care to accomplish the 2030 goal.

## METHODS

### Data sources

This is a descriptive observational analysis with data extraction from GBD 2019 data set using Global Health Data Exchange (GHDx, https://ghdx.healthdata.org). GBD 2019 incorporated nationally representative surveys, censuses, and meta-analysis results to estimate the incidence, prevalence, mortality, years of life lost (YLLs), years lived with disability (YLDs), and disability-adjusted life-years (DALYs) for 369 diseases and injuries in 204 countries and territories [13]. The overall GBD 2019 methodologies have been detailed previously. In this study, six indicators (incidence, prevalence, mortality, DALY, YLL, YLD) of cervical cancer were collected from GBD 2019 data set. Data on the coverage of cervical cancer screening were obtained from the WHO Global Health Observatory (https://www.who.int/data/gho) [14].

### Quality of Care Index (QCI)

First, four secondary indicators were constructed based on the age-standardised rate retrieved from the GBD 2019 data set, namely (1) ratio of YLLs to YLDs, (2) ratio of DALYs to prevalence, (3) mortality-to-incidence ratio, and (4) prevalence-to-incidence ratio. Then the QCI was determined using principal component analysis (PCA) based on the above four secondary indicators. PCA involved mathematical multivariate analysis to extract linear combinations as orthogonal components of the above four secondary indicators. The first component that best described the variance and variability in the data are denoted the QCI and allocated a score of 0-100. Higher scores on the QCI indicated a high quality of cervical cancer care [15-17].

Ratio of YLLs and YLDs = YLLs / YLDs

Ratio of DALYs to prevalence = DALYs / Prevalence

Mortality-to-incidence ratio = Mortality / Incidence

Prevalence-to-incidence ratio = Prevalence / Incidence

### Statistical analysis

In this study, the disease burden and trend of cervical cancer from 1990 to 2019 were analysed. Then QCI was constructed and the disparity of QCI across regions, countries, and age groups was assessed. Cervical cancer QCI was also evaluated in each socio-development quintile according to the sociodemographic index (SDI). The SDI is a geometric average of zero to one in each country or region. The SDI is obtained by combining the total fertility rate of women younger than 25 years, the education level of people aged 15 years and older, and the lag in the per capita income distribution. Countries and regions were divided into five levels according to the SDI, as follows: high (>0.81), high-middle (0.70-0.81), middle (0.61-0.69), low-middle (0.46-0.60), and low (<0.46) [18].

To explore the association between cervical cancer screening and the quality of cervical cancer care, we constructed a linear regression model, with cervical cancer QCI as the dependent variable and cervical cancer screening coverage as the independent variable. The association were further conducted in low development countries and high development countries, separately. We also used sensitivity analysis to test the robustness of the results, with prevalence of women screened for cervical cancer in the last year, in the last five years, and in the last three years used to represent screening coverage in the multiple regression model. The four secondary indicators were also used as the outcome variable in logistic regression model to identify the robustness of the results.

All statistical analysis in this study were conducted using STATA (Version 14.0; Stata Corp LLC, TX, USA) and R v4.1.3 software (http://www.r-project.org/). All tests were two-sided and *P*-values <0.05 were considered statistically significant.

## RESULTS

### Disease burden of cervical cancer

In 2019, cervical cancer caused a total of 280 479.04 deaths worldwide, 8 955 012.78 DALYs, and the corresponding age-standardised deaths and DALY rates reached 6.51 and 210.64 per 100 000 women-years, respectively. In different SDI regions, the disease burdens of death and DALY were the highest in the middle SDI region, and the age-standardised rate was the highest in the low-middle region. From 1990 to 2019, the age-standardised rate showed a decreasing trend globally and in all SDI regions, while the absolute value of cervical cancer disease burden still showed an increase due to population growth (**Table 1**). The age distribution of cervical cancer disease burdens showed an inverse U shape and reaching its peak at the age of 40-44 for prevalence and at the age of 50-59 for DALY, YLL and YLD. Death and DALYs of cervical cancer in 35-59 years of age accounted for half of all cancer burdens. The data are presented in Table S1 in the **Online Supplementary Document**.

**Table 1:** All-age numbers and age-standardised rate of cervical cancer in 1990 and 2019, by sociodemographic index (SDI) regions

Disease burden	Global		High SDI		High-middle SDI		Middle SDI		Low-middle SDI		Low SDI	
	**Numbers**	**ASR**	**Numbers**	**ASR**	**Numbers**	**ASR**	**Numbers**	**ASR**	**Numbers**	**ASR**	**Numbers**	**ASR**
**Year of 1990**												
Deaths	184 527.08	8.48	25 222.04	4.56	41 352.74	6.95	52 526.26	11.71	39 209.43	19.18	26 080.41	9.32
DALYs	6 176 248.03	275.05	725 847.17	143.23	1 274 571.58	215.21	1 790 628.82	381.90	1 419 285.55	630.59	961 195.75	287.82
YLDs	134 770.32	5.90	27 492.66	5.60	30 883.21	5.22	36 213.55	6.51	25 060.91	9.50	15 017.73	5.58
YLLs	6 041 477.71	269.15	698 354.51	137.63	1 243 688.36	209.99	1 754 415.27	375.39	1 394 224.64	621.09	946 178.02	282.23
Prevalence	1 538 196.47	66.20	341 394.21	71.39	353 661.58	60.00	409 752.89	67.49	27 4901.60	92.71	157 298.55	60.08
Incidence	335 641.56	14.91	59 690.30	11.83	75 804.79	12.77	92 178.41	18.04	66 215.52	27.74	41 498.75	14.87
**Year of 2019**												
Deaths	280 479.04	6.51	26 173.04	2.90	51 771.14	4.89	90 099.72	8.85	66 677.78	15.05	45 540.45	6.78
DALYs	8 955 012.78	210.64	672 113.02	89.72	1 543 704.05	154.69	2 817 246.13	285.64	2 282 244.87	477.53	1 632 489.70	204.60
YLDs	242 050.70	5.75	30 516.53	4.46	50 782.48	5.32	78 743.30	6.29	51 403.96	8.43	30 423.12	5.72
YLLs	8 712 962.08	204.89	641 596.49	85.26	1 492 921.57	149.36	2 738 502.82	279.35	2 230 840.91	469.09	1 602 066.58	198.89
Prevalence	288 7521.23	69.10	377 169.44	58.42	61 8791.45	66.79	946 469.33	71.74	601 452.10	88.05	341 465.18	68.38
Incidence	565 540.89	13.35	63 864.06	8.91	113 123.01	11.59	183 337.34	15.78	125 962.83	23.21	78821.30	13.44

ASR – age-standardised rate, DALY – disability-adjusted life-years, SDI – socio-demographic index, YLD – years lived with disability, YLL – years of life lost

### Cervical cancer QCIs

**Figure 1:**
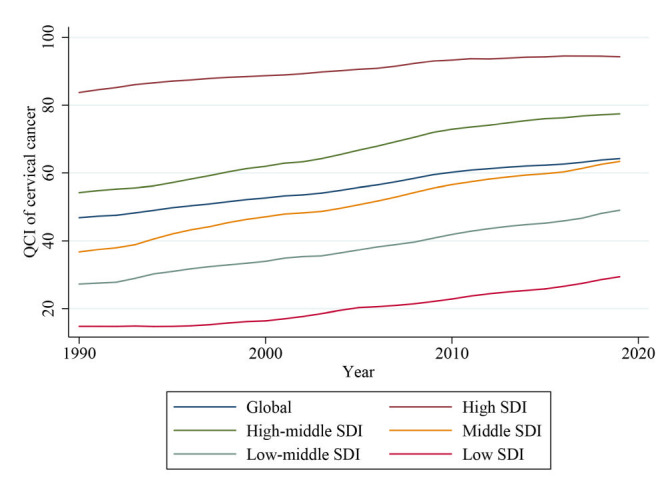
Trend of Quality of Care Index (QCI) for cervical cancer from 1990 to 2019, by sociodemographic index (SDI) regions.

In 2019, the global average level of cervical cancer QCIs was 64.21. From 1990 to 2019, the global cervical cancer QCI increased year by year, and it increased by 37.11% in 2019 compared with 1990. Specifically, QCI grew rapid from 1990 to 2009, and slower from 2009 to 2019 (**Figure 1** and Table S2 in the **Online Supplementary Document**).

**Figure 2:**
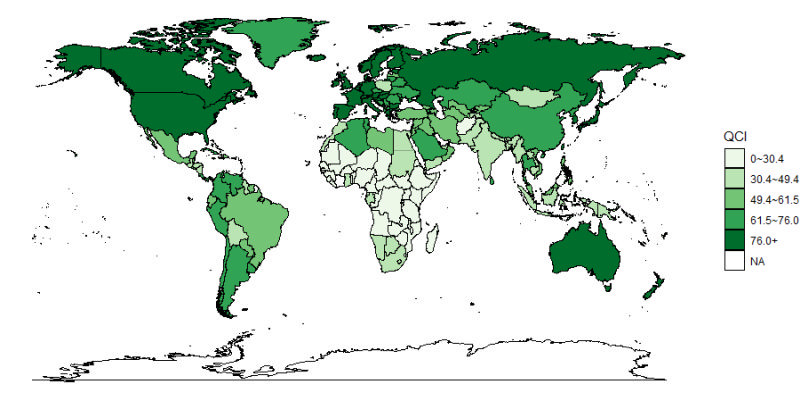
Global map of Quality of Care Index (QCI) for cervical cancer in 2019.

Regional and national disparity existed in the cervical cancer QCI as well as the time trend. Highest QCI occurred in High-income Asia Pacific and reaching 97.02; the lowest QCI was in Central Sub-Saharan Africa and holding at 23.20. Among the 21 GBD regions, cervical cancer QCI grew fastest in East Asia from 1990 to 2019, with an increasement of about 1.5 times in 30 years, and the slowest growth was in Southern Sub-Saharan Africa, with an increase of only 2.15% in 30 years (Table S3 in the **Online Supplementary Document**). At national level, Canada has the highest cervical cancer QCI of 99.98, whereas Somalia has the lowest QCI of 0.10. The distribution of cervical cancer QCIs among different countries is as shown in **Figure 2**.

Cervical cancer QCI showed an unbalanced pattern across regions with development levels. Cervical cancer QCI was positively associated with social development status, that QCI in high and high-middle SDI countries surpassed the global average level, QCI in middle SDI countries were around the global average, whereas countries in low-middle and low SDI had an inferior QCI. From 1990 to 2019, the cervical cancer QCIs grew fast in the countries with high-middle and middle SDI, the estimated annual percentage changes (EAPC) of which were 0.90 (95% confidence interval (CI) = 0.86-0.94) and 0.92 (95% CI = 0.90-0.95) per year, respectively. However, the cervical cancer QCI grew slowly, which was only 0.53 (95% CI = 0.49-0.57). The above results indicate that the difference in the quality of cervical cancer care between low-income regions and other regions may expand in the future.

**Figure 3:**
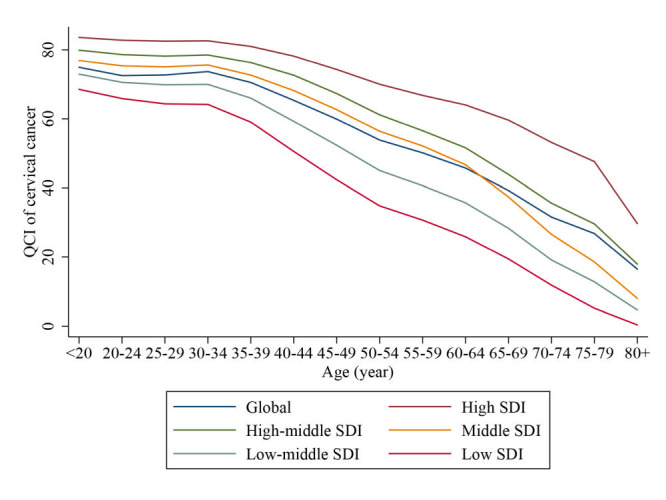
Trend of Quality of Care Index (QCI) for cervical cancer with age group, by sociodemographic index (SDI) regions.

At the global level, the cervical cancer QCI showed a downward trend with age (**Figure 3**). With the age of 35 as the cut-off point, the cervical cancer QCI was relatively stable for the women under the age of 35, but dropped rapidly after the age of 35. This pattern was consistent across all SDI regions with the decreasing trend after 35 years of age.

### Cervical cancer screening and QCI

**Figure 4:**
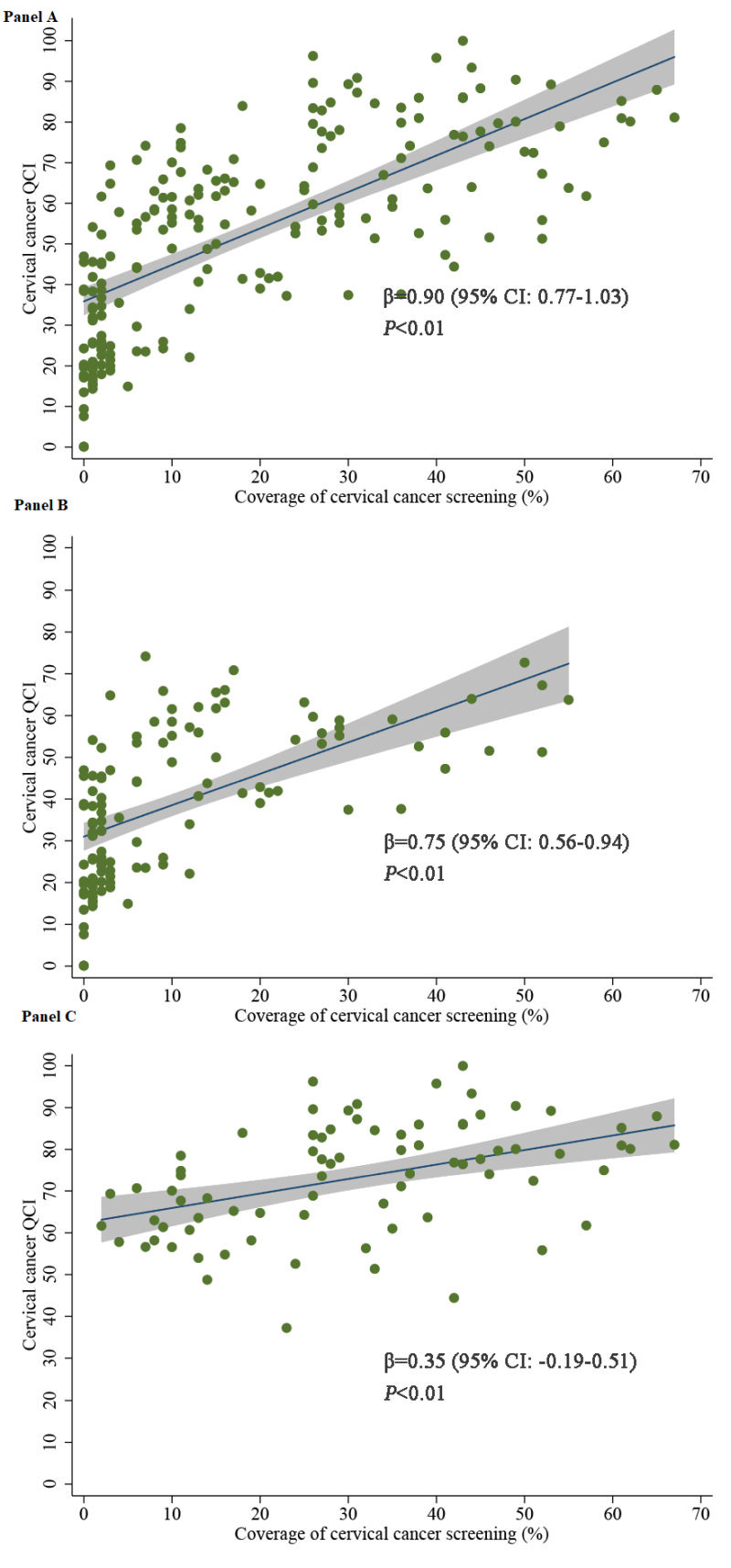
Association between cervical cancer screening coverage and Quality of Care Index (QCI). **Panel A.** The association in all 194 countries. **Panel B.** The association in 116 countries with a low development level (low, middle-low and middle sociodemographic index (SDI)). **Panel C.** The association in 78 countries with a high development level (high-middle and high SDI).

The association between cervical cancer screening coverage and cervical cancer QCI was evaluated across 194 countries. Results in **Figure 4** showed that cervical cancer screening coverage was positively associated with cervical cancer QCI. For every percent increase in the cervical cancer screening coverage, the cervical cancer QCI will increase by 0.90 (95% CI = 0.77-1.03, *P* < 0.01). This association followed different patterns across countries with different development levels. In the countries with a low development level (low, low-middle and middle SDI), better cervical cancer QCI was strongly correlated with high screening coverage (β = 0.75, 95% CI = 0.56-0.94, *P* < 0.01), while the correlation was weaker (β = 0.35, 95% CI = 0.19-0.51, *P* < 0.01) in countries with a high development level (high-middle and high SDI).

Sensitivity analysis showed consistent results by using the indicator of three-year, five-year, and lifetime cervical cancer screening coverage, as well as using the four secondary indicators as the outcome variable. Related results (Table S4 and Table S5 in the **Online Supplementary Document**) indicated that providing the cervical cancer screening coverage can significantly improve the quality of cervical cancer care, and this association was more significant in countries with low and middle development levels.

## DISCUSSION

Good quality of cervical cancer care was crucial to achieve the global goal of cervical elimination. This study found that the quality of cervical cancer care was unbalanced among regions with different development levels and the disparity between low-income regions and other regions may expand. A higher cervical cancer screening coverage was correlated with better QCI, and this association was stronger in countries with low- and middle-development levels. The relevant research results were supportive to identify vulnerable regions and populations, and to provide evidence for guidance of further improvement of global prevention and control strategies for cervical cancer.

The quality of cervical cancer care was unbalanced among regions with different development levels, and the growth rate was slower in the regions with a low SDI, suggesting that the global inequality in the quality of cervical cancer care may be further intensified in the future. The reasons for the regional-level inequality were complex. First, all cervical cancer prevention strategies required resources like well-trained manpower, appropriate equipment, and situation-adapted technology, which was limited and insufficient for low and middle-development countries [19-21]. For example, low-income countries were of limited financial resources and infrastructure to carry out population-based screening programs, which could detect cervical cancer at early stage and improve the quality of cervical cancer care [22]. Second, population-level compliance was also of disparity across regions. A literature review shows that the population compliance of cervical cancer screening in high-income countries is more than three times that of low- and middle-income countries [23]. The compliance in European region (higher than 80%) is much higher than that in African region (only 6%) on average [23]. The social determinants of health [24-26], like poor health literacy, insufficient awareness of the benefits of screening, low education, and cultural and religious barriers, could lead to low-level acceptance of key cervical cancer prevention and control measures in general population, which would exacerbate the regional disparity in quality of cervical cancer care.

Cervical cancer QCI was of disparity across age groups. In this study, we found that the cervical cancer QCI was relatively stable for the women under the age of 35, but drop rapidly after the age of 35. With the global strategy proposed by the WHO regards women aged 30-49 years as a priority in cervical cancer elimination, then the management after screening seemed to be the key to improve cervical cancer care quality. WHO guidelines also highlighted the need to provide timely and effective treatment and management of cervical pre-cancer lesions. International Agency for Research on Cancer position paper [27] highlighted the need to provide adequate follow-up, triage, and appropriate treatment for women with positive screening results, which was an important prerequisite for successful cervical cancer prevention and was also the major issue and challenge currently for countries with limited resources and low- and middle-development status.

A positive association between cervical cancer screening coverage and QCI identified in this study indicated that screening was an important strategy to improve cervical cancer quality of care, especially in low and middle development countries. Epidemiological studies proved that cervical cancer screening could detect cervical cancer patients at early stage and reduce the cervical cancer mortality [28]. Then screening and early detection may bring the opportunity to offer timely access to care and appropriate treatment to patients detected at early stage, which could be the mainstay of the association. Cervical cancer screening and effective post-screening management are important components of the global strategy to eliminate cervical cancer designated by the WHO, and it is required that 70% screening coverage with a high-performance test and 90% of women with a positive screening test or a cervical lesion managed appropriately to achieve the 2030 goal [11]. The positive association between cervical cancer screening and the quality of cervical cancer care found in this study further supported the importance of implementation of appropriate cervical cancer screening strategies, especially in the countries with a low development level, which is of great significance to promote the improvement of health outcomes.

This study has several strengths and advantages. QCI index constructed in this analysis could present the current global landscape of quality of cervical cancer care, and to inform possible strategies to improve the QCI. This study also has limitations. QCI was calculated based on country-level data applying PCA analysis, which showed the averaging effect and may ignore specific subgroup characteristics. Second, the QCI defined in this study focused on the quality of cancer care by synthesising the incidence, prevalence, mortality, DALYs, YLLs and YLDs, which may be biased with undiagnosed cancers. Third, we did not explore the association between cervical cancer vaccination and quality of cervical cancer care in this study, based on the assumption that vaccination could reduce the long-term cervical cancer incidence rather than improve the cancer care quality.

## CONCLUSIONS

In summary, this study informed that the quality of cervical cancer care was disparate across regions and populations. Population in low-development countries and the 35-39 age group were vulnerable to access high-quality cervical cancer care. Higher screening coverage was associated with better cervical cancer QCI, implying that expanding cervical cancer screening coverage may be an effective strategy to improve the quality of cervical cancer care.

## Additional material


Online Supplementary Document
