# Supplementary material for: The association of cervical cancer screening and quality of care: A systematic analysis of the Global Burden of Disease Study 2019
Source: J Glob Health. 2023 Aug 25;13:04090. doi: 10.7189/jogh.13.04090 (PMC10451102; doi:10.7189/jogh.13.04090)
Supplement: Online Supplementary Document [file jogh-13-04090-s001.pdf]

Supplementary information

Title: The Association of Cervical Cancer Screening and Quality of Care: A Systematic Analysis of the Global Burden of Disease Study 2019

Minmin Wang, Ph.D. [1,2], Yinzi Jin, Ph.D. [1,2*], Zhi-Jie Zheng, M.D., Ph.D. [1,2]

1 Department of Global Health, School of Public Health, Peking University, Beijing, China
2 Institute for Global Health and Development, Peking University, Beijing, China

*Correspondence to:
Yinzi Jin, PhD, Department of Global Health, School of Public Health, Peking University, 38 Xue Yuan Road, Haidian District, Beijing 100191, China
E-mail: yzjin@bjmu.edu.cn
Fax number: +8610-82802144
Telephone number: +86-15001081989

Table of Contents

Table S1. All-age numbers and age-standardized rate of Cervical Cancer in 2019, by age groups.

Table S2. The Quality of Care Index for cervical cancer from 1990 to 2019, by SDI regions.

Table S3. The Quality of Care Index and percentage of change for cervical cancer by regions.

Table S4. Sensitivity analysis of the association between cervical cancer screening coverage and quality of care index.

Table S5. Association analysis of cervical screening coverage and four secondary indicators.

Table S1. All-age numbers and age-standardized rate of Cervical Cancer in 2019, by age groups.

Age	Deaths		DALYs		YLDs		YLLs		Prevalence		Incidence	
	Number	Rate	Number	Rate	Number	Rate	Number	Rate	Number	Rate	Number	Rate
<20 years	235.89	0.02	17575.04	1.41	722.71	0.06	16852.33	1.35	10639.81	0.85	1360.47	0.11
20-24 years	1141.79	0.39	78892.13	26.67	2903.92	0.98	75988.22	25.69	43351.09	14.66	5737.32	1.94
25-29 years	3450.69	1.15	221014.54	73.50	8453.41	2.81	212561.12	70.69	125614.48	41.77	16745.78	5.57
30-34 years	8198.48	2.75	484444.64	162.26	20240.37	6.78	464204.27	155.48	297075.22	99.50	39556.90	13.25
35-39 years	14141.15	5.27	758614.42	282.61	28184.07	10.50	730430.35	272.11	401001.54	149.38	55857.98	20.81
40-44 years	21987.15	8.99	1061376.62	433.75	33235.58	13.58	1028141.03	420.17	452156.74	184.78	68350.78	27.93
45-49 years	27394.34	11.64	1179324.33	500.93	32351.51	13.74	1146972.82	487.18	412337.35	175.14	69292.94	29.43
50-54 years	34625.99	15.79	1317791.88	601.10	32154.87	14.67	1285637.01	586.43	375419.64	171.24	72410.53	33.03
55-59 years	34911.48	18.55	1159591.31	615.98	27180.82	14.44	1132410.49	601.54	299510.83	159.10	64990.54	34.52
60-64 years	31729.48	19.78	903278.00	563.21	20245.68	12.62	883032.32	550.59	206685.32	128.87	52614.49	32.81
65-69 years	29517.60	21.87	703586.31	521.30	14703.99	10.89	688882.32	510.41	131391.03	97.35	42404.81	31.42
70-74 years	24987.04	25.24	486364.94	491.36	9600.98	9.70	476763.96	481.66	70280.52	71.00	30653.90	30.97
75-79 years	18870.77	27.02	290150.51	415.40	5856.75	8.38	284293.75	407.01	35875.90	51.36	20481.08	29.32
80+ years	29287.18	32.03	293008.11	320.48	6216.02	6.80	286792.08	313.68	26181.77	28.64	25083.36	27.43

DALY, disability-adjusted life-years; YLD, years lived with disability; YLL, years of life lost.

Table S2. The Quality of Care Index for cervical cancer from 1990 to 2019, by SDI regions.

Year	Global	High SDI	High-middle SDI	Middle SDI	Low-middle SDI	Low SDI
**1990**	46.83	83.74	54.23	36.71	27.24	14.84
**1991**	47.28	84.53	54.80	37.42	27.51	14.83
**1992**	47.55	85.20	55.25	37.97	27.79	14.82
**1993**	48.26	86.07	55.59	38.89	28.93	14.93
**1994**	48.99	86.58	56.18	40.57	30.23	14.79
**1995**	49.78	87.09	57.15	42.06	30.95	14.83
**1996**	50.35	87.44	58.20	43.27	31.74	14.98
**1997**	50.89	87.89	59.21	44.20	32.38	15.32
**1998**	51.55	88.20	60.31	45.40	32.88	15.83
**1999**	52.18	88.44	61.28	46.36	33.38	16.23
**2000**	52.65	88.69	61.94	47.10	33.94	16.44
**2001**	53.25	88.91	62.85	47.91	34.87	17.04
**2002**	53.55	89.28	63.27	48.26	35.35	17.73
**2003**	54.08	89.78	64.20	48.68	35.54	18.57
**2004**	54.86	90.15	65.40	49.59	36.41	19.50
**2005**	55.74	90.57	66.70	50.66	37.31	20.30
**2006**	56.48	90.87	67.88	51.73	38.22	20.58
**2007**	57.40	91.51	69.21	52.93	38.92	20.94
**2008**	58.43	92.33	70.53	54.27	39.67	21.44
**2009**	59.48	93.04	71.95	55.55	40.80	22.10
**2010**	60.19	93.34	72.86	56.56	41.91	22.83
**2011**	60.82	93.74	73.51	57.38	42.85	23.70
**2012**	61.24	93.67	74.05	58.18	43.62	24.38
**2013**	61.68	93.91	74.80	58.80	44.29	24.93
**2014**	62.03	94.19	75.46	59.37	44.82	25.36
**2015**	62.27	94.28	76.03	59.76	45.23	25.81
**2016**	62.60	94.54	76.28	60.29	45.91	26.57
**2017**	63.13	94.53	76.82	61.34	46.72	27.45
**2018**	63.79	94.50	77.17	62.51	48.09	28.54
**2019**	64.21	94.32	77.44	63.37	49.05	29.39
**EAPC**	0.64 (0.62-0.66)	0.38 (0.35-0.40)	0.90 (0.86-0.94)	0.92 (0.90-0.95)	0.75 (0.73-0.77)	0.53 (0.49-0.57)

EAPC, estimated annual percentage changes; SDI, socio-demographic index.

Table S3. The Quality of Care Index and percentage of change for cervical cancer by regions.

Location	Cervical cancer QCI in 1990	Cervical cancer QCI in 2019	Percentage of Change (%)
**Global**	44.26	60.31	136.27
**Andean Latin America**	36.47	63.46	174.02
**Australasia**	88.54	96.95	109.50
**Caribbean**	43.83	57.42	131.01
**Central Asia**	52.21	62.20	119.14
**Central Europe**	59.18	72.35	122.26
**Central Latin America**	35.08	60.04	171.15
**Central Sub-Saharan Africa**	12.43	23.20	186.63
**East Asia**	27.05	66.53	245.99
**Eastern Europe**	54.76	77.82	142.11
**Eastern Sub-Saharan Africa**	13.27	25.86	194.93
**High-income Asia Pacific**	82.70	97.02	117.32
**High-income North America**	81.75	85.74	104.87
**North Africa and Middle East**	35.50	54.47	153.44
**Oceania**	34.84	41.66	119.59
**South Asia**	24.01	44.88	186.89
**Southeast Asia**	40.51	58.56	144.58
**Southern Latin America**	62.45	76.77	122.93
**Southern Sub-Saharan Africa**	41.50	42.39	102.15
**Tropical Latin America**	39.89	61.58	154.37
**Western Europe**	78.00	89.21	114.36
**Western Sub-Saharan Africa**	17.65	27.80	157.50

Table S4. Sensitivity analysis of the association between cervical cancer screening coverage and quality of care index.

Indicator of cervical cancer screening coverage	Global β (95% CI)	Low, low-middle and middle SDI β (95% CI)	High-middle, high SDI β (95% CI)
**Coverage in last year**	0.90 (0.77-1.03)	0.75 (0.56-0.94)	0.35 (0.19-0.51)
**Coverage in last 3 years**	0.61 (0.54-0.68)	0.48 (0.37-0.59)	0.30 (0.19-0.41)
**Coverage in last 5 years**	0.56 (0.49-0.62)	0.43 (0.33-0.53)	0.28 (0.17-0.39)
**Coverage in lifetime**	0.50 (0.44-0.56)	0.38 (0.29-0.46)	0.25 (0.15-0.36)

Table S5. Association analysis of cervical screening coverage and four secondary indicators.

Secondary Indicators	Global β (95% CI)	Low, low-middle and middle SDI β (95% CI)	High-middle, high SDI β (95% CI)
**Ratio of YLLs to YLDs**	-0.51 (-0.59, -0.43)	-0.44 (-0.55, -0.32)	-0.16 (-0.25, -0.08)
**Ratio of DALYs to incidence**	-0.06 (-0.06, -0.05)	-0.05 (-0.07, -0.04)	-0.02 (-0.02, -0.01)
**Mortality-to-incidence ratio**	-0.01 (-0.01, -0.003)	-0.004 (-0.004, -0.003)	-0.002 (-0.003, -0.001)
**Prevalence-to-incidence ratio**	0.04 (0.03, 0.05)	0.03 (0.02, 0.04)	0.02 (0.01, 0.03)
